# Hepatitis in Malaysia: Past, Present, and Future

**DOI:** 10.5005/jp-journals-10018-1167

**Published:** 2016-07-09

**Authors:** Ruksana Raihan

**Affiliations:** Faculty of Medicine, Aimst University, Kedah, Malaysia

**Keywords:** Future, Hepatitis, Malaysia, Past, Present.

## Abstract

Malaysia is multiethnic, with a population of 31,127,247 comprising a mixture of Malays (50.1%), Chinese (22.6%), Indians (6.7%), Aborigines (11.8%), others (0.7%), and noncitizens (8.2%). Like other countries in the region, viral hepatitis is an important public health problem in Malaysia. The 3 most common causes for hepatitis in Malaysia are hepatitis A, B, and C. Hepatitis A has been a reportable disease in Malaysia since 1988. Due to the introduction of government control programs, the national incidence rate has dropped steadily. It is now estimated that 50% of Malaysians less than 30 years of age do not have antibodies to hepatitis A and are therefore susceptible to the disease, which can be prevented by reinforcing the hygiene status of the general population. Malaysia is a country of medium seroprevalence for the hepatitis B virus (HBV) surface antigen (HBsAg) in the general population (1.5-9.8%). The major route of transmission is from infected mother to fetus. There are an estimated 1 million people chronically infected with hepatitis B in Malaysia. Approximately 75% of all viral hepatitis cases are due to hepatitis B infection, with a male-to-female ratio of 2:1. Chronic hepatitis B (CHB) accounts for more than 80% of the hepatocellular carcinoma (HCC) cases seen in Malaysia and HCC is the 3rd most common malignant neoplasm and among the 10 leading causes of death. Most common genotypes are B and C. Incidence rates among Chinese, Malays, and Indians are 36, 26, and 15% respectively. The hepatitis B vaccination program for children was introduced in 1989, which successfully managed to reduce the seroprevalence of infection among Malaysians to 0.01% (graph 4, 2014). But the disease burden will still remain high for some time as the infected people are getting older and living longer. Hepatitis C virus (HCV) infection is a growing problem in Malaysia. An estimated 453,700 people were living with HCV infection in Malaysia in 2009 (2.5% of the population aged 15-64 years), of whom 59% acquired their infection through injection and the most common genotypes found are genotype 3 and 1. The HCV-related disease burden is already high and is forecast to rise steeply over the coming decades under current levels of antiviral treatment. Increased governmental resources to improve HCV screening and treatment rates and to reduce transmission are essential to address the high projected HCV disease burden in Malaysia.

**How to cite this article:**

Raihan R. Hepatitis in Malaysia: Past, Present, and Future. Euroasian J Hepato-Gastroenterol 2016;6(1):52-55.

## INTRODUCTION

**Graph 1:**
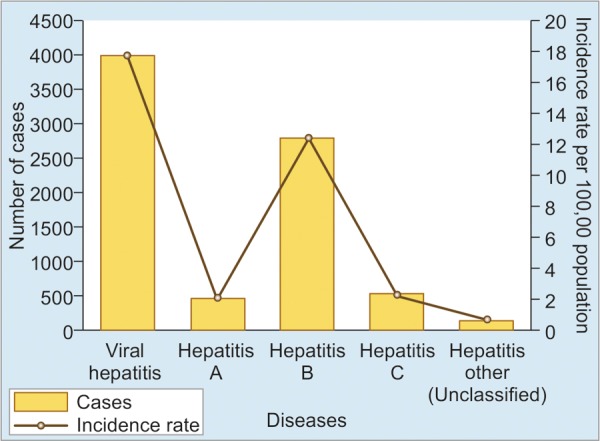
Symptomatic viral hepatitis in 2000

Malaysia is a federal country located in Southeast Asia. It consists of 13 states and 3 federal territories and has a total landmass of 329,847 km^2^ (127,350 sq mi) separated by the South China Sea into 2 similarly sized regions: Peninsular Malaysia and East Malaysia (Malaysian Borneo). Peninsular Malaysia is home to 79.3% of the entire population – about 4 times more populated than East Malaysia. The country is multiethnic, with a population of 31,127,247 comprising a mixture of Malays (50.1%), Chinese (22.6%), Indians (6.7%), Aborigines (11.8%), others (0.7%), and noncitizens 8.2%. Like other countries in the region, viral hepatitis is an important public health problem in Malaysia. The 3 most common causes for hepatitis in Malaysia are hepatitis A, B, and C. During the year 2000, approximately 4,067 cases of viral hepatitis were seen in Malaysia. Of this, approximately 497 were due to hepatitis A, 2,863 due to hepatitis B, and 550 due to hepatitis C (Graph 1).^1^

## PAST AND PRESENT

### Hepatitis A

**Graph 2:**
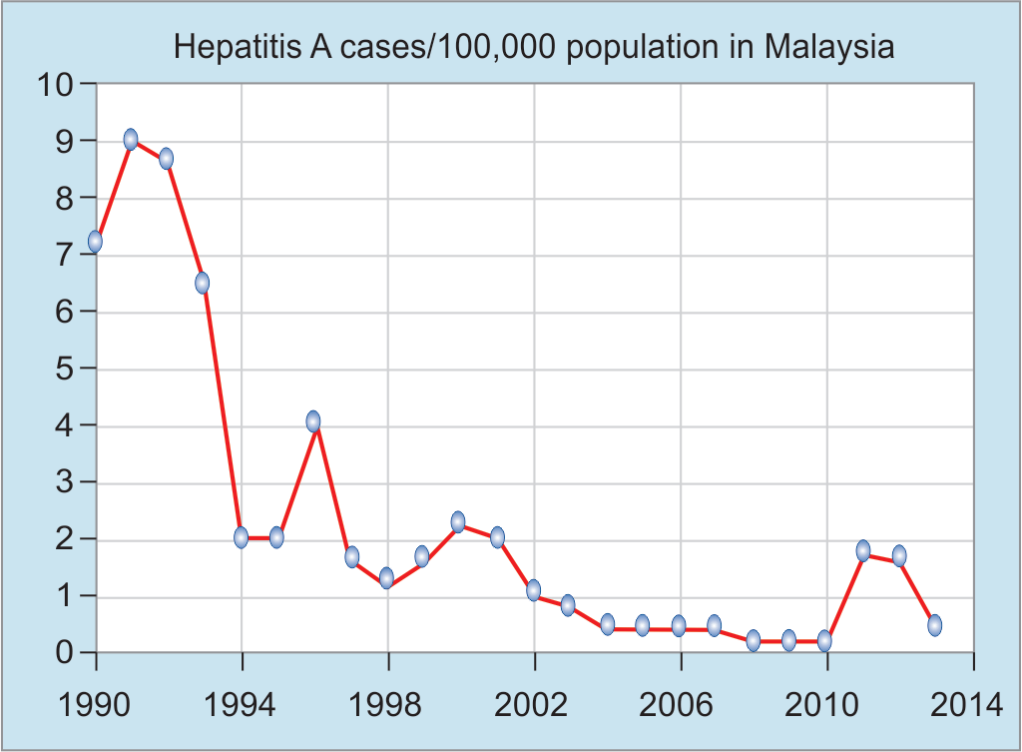
Hepatitis A virus seroprevalence from 1990 to 2014

Hepatitis A has been a reportable disease in Malaysia since 1988. Results from seroepidemiology studies carried out over the past decade have shown that due to the introduction of government control programs, the national incidence rate had dropped steadily from 2.24/100,000 population in 2000 to 0.41/100,000 in 2013 (Graph 2).^1,2^ Hepatitis A virus (HAV) has been reported to be the main cause of symptomatic clinical hepatitis in up to 66.4% cases in 1996 in the eastern region of Peninsular Malaysia when compared to other causes of viral hepatitis, whereas that figure came down to only 12.22% in the year 2000.^3^ A study by Ton et al^4^ investigated 100 healthy individuals from Kuala Lumpur in 1983 and reported a seroprevalence of hepatitis A of 78.2%. In 1985, a study reported that 100% of Malaysian population were anti-HAV positive by 30 years of age. However, in 1992, only 45% of the same age group was antibody positive, indicating a shifting epidemiology, most probably due to improvements in living standards. The prevalence of HAV in Malaysia as expected is falling with time; this means, however, that reintroduction of the virus to a nonimmune population could produce a community-level outbreak, which may lead to an increase in morbidity and mortality.^5^ Few notable outbreaks have been reported in the last 15 years. An outbreak (51 cases) was reported in the Hulu Langat district from April to October 2002 and the possible cause was river water fecal contamination. Another outbreak (800 cases) was reported in Terengganu state in the year 2011 and was associated with contaminated rain water. In October 2012, 78 people who drank toddy were affected with hepatitis A in Manjung, Perak.

### Hepatitis B

Malaysia is a country of medium seroprevalence for hepatitis B virus (HBV) surface antigen (HBsAg) in the general population (1.5-9.8%). Most HBV carriers are infected prenatally owing to the high viral load in Malaysian women of child-bearing age. An estimated 1 million people are chronically infected with hepatitis B in Malaysia. Approximately 75% of all viral hepatitis cases are due to hepatitis B infection, with a male-to-female ratio of 2:1. Chronic hepatitis B (CHB) accounts for more than 80% of the hepatocellular carcinoma (HCC) cases seen in Malaysia, and HCC is the 3rd most common malignant neoplasm and among the 10 leading causes of death. Most common genotypes are B and C. Incidence rates among Chinese, Malays, and Indians are 36, 26, and 15% respectively. The prevalence of HBV among HIV-positive individuals in a tertiary care hospital is 13% (2014).^6^

**Graph 3:**
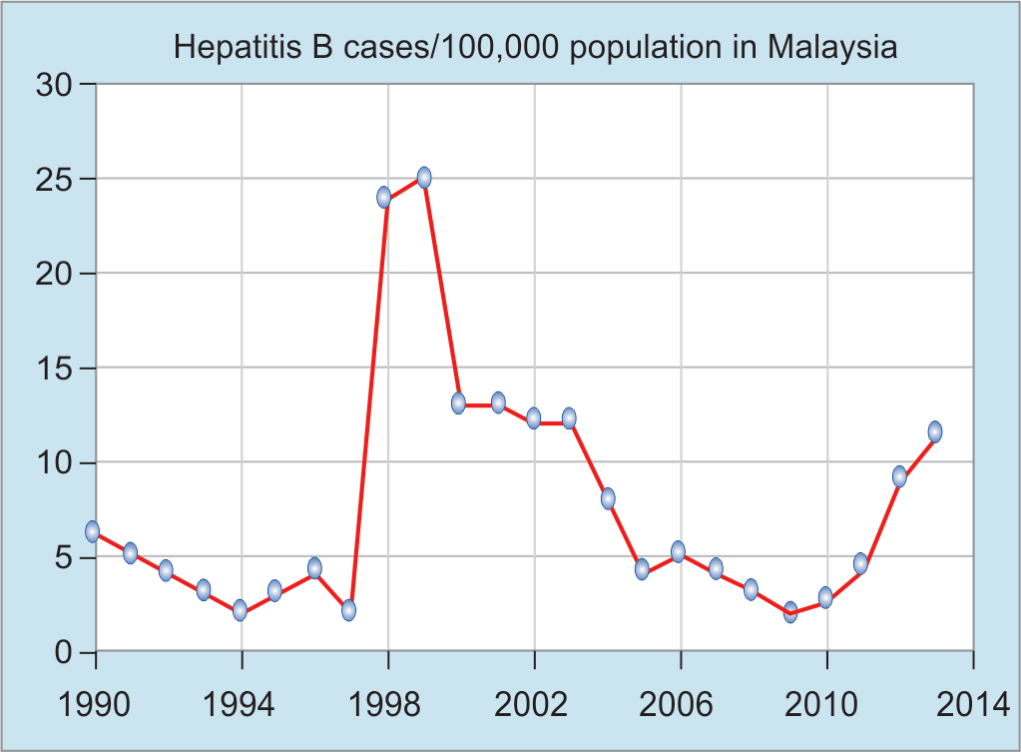
Hepatitis B virus prevalence in Malaysia

The incidence rate of HBV in Malaysia from 1990 to 2013 demonstrated a steady decrease from 1990 to 1997 due to the universal vaccination of infants, which commenced in 1989, and various control programs (Graph 3). In 1998, however, a sharp increase in reported cases was noted due to the inclusion of mandatory testing of all foreign workers who had arrived in Malaysia. The decrease trend followed after 2000, which rose again from 2010, as government has implemented the rule that all cases of hepatitis B be reported to hospitals.^2^

The national hepatitis B immunization coverage in infant is 96.32% (2013).^2^ The impact of universal immunization program on the prevalence of CHB infection in the Malaysian population has been assessed by Ng et al for 190,077 school children aged 7 to 12 years from 1997 to 2003. The HBsAg detection rate was 2.5% among those born in 1985 (before the implementation of universal infant vaccination), which declined to 0.4% among those born in 1996.^7,8^ In another study among the 2,923 new student intakes in the Faculties of Medicine and Dentistry, University of Malaya, from 2005 to 2011, the overall HBsAg prevalence was 0.62%. The HBsAg prevalence rate was 1.08% (15/1,390) among those born before 1989 and only 0.20% (3/1,533) among those born in or after 1989. HBsAg prevalence declined steadily from 1.27% (5/394) in 2005 to 1.20% (5/418) in 2006, 0.95% (4/421) in 2007, 0.49% (2/410) in 2008, 0.49% (2/407) in 2009, and finally 0% in both 2010 (0/445) and 2011 (0/428). This trend attested the efficacy of the universal infant HBV vaccination program for protection against chronic HBV infection, for at least 18 to 19 years, without a booster vaccination.^8^

### Hepatitis C

**Graph 4:**
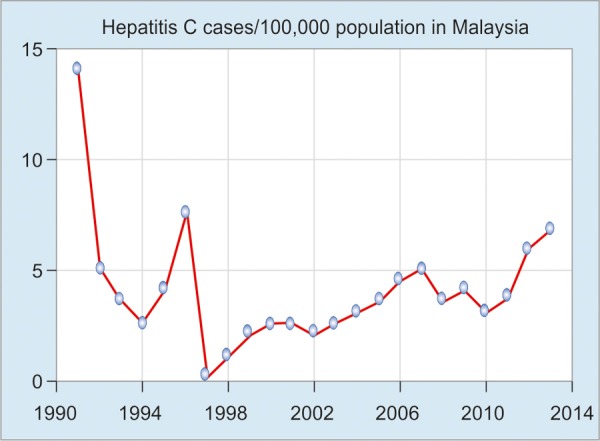
Hepatitis C virus seroprevalence from 1990 to 2014

Hepatitis C virus (HCV) infection is a growing problem in Malaysia as more and more people are found to have antibodies to HCV through routine screening. An estimated 453,700 people were living with HCV infection in Malaysia in 2009 (2.5% of the population aged 15-64 years), of whom 59% acquired their infection through injection. Yet awareness about hepatitis C in Malaysia is low.^9^ In 2000, there were 550 reported cases of hepatitis C, with an incidence rate of 2.5/100,000 population; in 2004 there were 741 cases with an incidence rate of 2.9/100,000 population, and in the year 2013 the incidence rate became 6.77/100,000, which reflects the overgrown disease burden (Graph 4).^2^

The most common genotypes found in Malaysia are genotype 3 and 1. Many studies showed that over the past 15 years this genotype distribution remain unchanged in Malaysia. The estimated prevalence in females is considerably lower than among males, and the estimated number of HCV antibody-positive male Malays are much higher (84%) than the numbers for the other 2 ethnicity groups. The main modes of HCV transmission identified were parenteral drug use (85%), transfusion (3%), and/or dialysis.^10,11^

## FUTURE

According to ministry records, 5% of Malaysians suffer from hepatitis B, while 2% carry HCV. Statistics also show that more than 80% of hepatitis B and C patients are aged between 25 and 55 years. The nationwide HBV vaccination program for children was introduced in 1989, which managed to reduce the rate of infection among Malaysians to 0.3% (exceeding WHO’s target of 1%). But the disease burden will still remain high for some time as the infected people are getting older and living longer.

The HCV-related disease burden is already high and is forecast to rise steeply over the coming decades under current levels of antiviral treatment. Although the current treatment practice is estimated to avert a cumulative total of 2,200 deaths from Liver cirrhosis (LC) or HCC, a cumulative total of 63,900 HCV-related deaths are projected by 2039 with a predicted 2,002 and 540 new decompensated cirrhosis (DC) cases and new HCC cases respectively. Increased governmental resources to improve HCV screening and treatment rates and to reduce transmission are essential to address the high projected HCV disease burden in Malaysia.^12^

## CONCLUSION

In summary, the prevalence of cirrhosis and the incidence of end-stage liver disease and death due to chronic HCV infection are projected to rise steeply over the next 25 years. A detailed economic analysis is required to estimate the implications of these burden projections for future public health expenditure associated with HCV infection. Increasing HBV and HCV screening and treatment rates, together with intensive efforts to reduce transmission (e.g., through vaccination, stepped-up harm reduction, education, and other prevention initiatives), would appear essential to address the existing HBV and predicted high HCV disease burden in Malaysia.
